# Autopsy‐Proven Immune‐Mediated Hepatitis With Rapid Liver Atrophy Despite Minimal Aminotransferase Elevation After Durvalumab Plus Tremelimumab Therapy for Hepatocellular Carcinoma: A Case Report

**DOI:** 10.1002/ccr3.72806

**Published:** 2026-05-26

**Authors:** Hidenobu Hara, Risa Katsumata, Ami Yoshinaga, Kotaro Amano, Hikari Ishii, Kei Onodera, Jyunko Nemoto, Ryota Nakamura, Harujiro Yamamoto, Shoichi Yokobori, Moe Yamashita, Hiroaki Matsumoto, Kazuomi Sakaki, Shiori Ito, Takehito Asakawa, Kouhei Yoshino, Jiro Kumagai, Shinya Sakita

**Affiliations:** ^1^ Department of Gastroenterology Yokohama City Minato Red Cross Hospital Yokohama Kanagawa Japan; ^2^ Department of Pathology Yokohama City Minato Red Cross Hospital Yokohama Kanagawa Japan

**Keywords:** autopsy, colitis, drug‐induced liver injury, hepatocellular carcinoma, immune checkpoint inhibitor

## Abstract

Immune‐mediated hepatitis caused by immune checkpoint inhibitors (ICIs) is typically suspected based on aminotransferase elevation; however, biochemical signals may be blunted in advanced cirrhosis, potentially delaying recognition. A 71‐year‐old woman with cirrhosis due to metabolic dysfunction–associated steatohepatitis and Barcelona Clinic Liver Cancer stage B hepatocellular carcinoma refractory to transarterial chemoembolization started durvalumab plus tremelimumab therapy. The patient developed grade 3 immune‐mediated colitis requiring high‐dose corticosteroids, with sustained clinical remission. Despite only modest aminotransferase changes, serial non‐contrast computed tomographic volumetry revealed progressive liver volume loss from 1.71 L pretreatment to 0.76 L at death, accompanied by jaundice and coagulopathy leading to fatal liver failure. In evaluating the subsequent liver dysfunction, the patient remained afebrile, and imaging revealed no biliary obstruction or vascular thrombosis. Autopsy revealed CD8‐predominant T‐cell infiltration with lobular hepatitis and patchy necrosis, consistent with immune‐mediated hepatitis as the cause of death. The tumor burden was small without metastasis. This case highlights that ICI hepatitis can progress with minimal aminotransferase elevation in advanced cirrhosis, warranting multidimensional monitoring beyond aspartate aminotransferase/alanine aminotransferase, including bilirubin, coagulation parameters, clinical decompensation, and quantitative imaging.

## Key Clinical Message

In patients with advanced cirrhosis, immune‐mediated hepatitis caused by immune checkpoint inhibitors may progress with minimal aminotransferase elevation. Serial non‐contrast CT volumetry quantified early, progressive liver volume loss, and autopsy confirmed CD8‐predominant lobular hepatitis as the cause of death. Monitoring beyond AST/ALT—bilirubin, coagulation, and quantitative imaging—may be warranted.

## 1 Introduction

Immune checkpoint inhibitors (ICIs) have become an established treatment option for advanced hepatocellular carcinoma (HCC), and durvalumab plus tremelimumab is one such regimen [1, 2, 3, 4, 5]. However, immune‐related adverse events can involve multiple organs, including the gastrointestinal tract and liver, and may be fatal in some cases [6, 7, 8]. Immune‐mediated hepatitis is typically suspected when aminotransferase levels increase, and severity assessment often relies primarily on aspartate aminotransferase/alanine aminotransferase (AST/ALT) level elevation [9, 10, 11]. In advanced cirrhosis, however, aminotransferase levels may underestimate the extent of hepatocellular injury, potentially delaying recognition of clinically significant immune‐mediated hepatitis and leading to life‐threatening outcomes.

Herein, we report a case of a patient with steatotic liver disease–related cirrhosis who developed immune‐mediated colitis requiring high‐dose corticosteroids after ICI therapy while concurrently experiencing rapidly progressive liver atrophy and fatal liver failure despite minimal aminotransferase elevation. Serial non‐contrast computed tomographic (CT) imaging and volumetry objectively quantified progressive liver volume loss over time.

## 2 Case History/Examination

A 71‐year‐old woman was referred for liver dysfunction in January 2018 and was diagnosed with cirrhosis attributed to nonalcoholic steatohepatitis (now termed metabolic dysfunction–associated steatohepatitis). On December 4, 2024, gadoxetic acid–enhanced magnetic resonance imaging showed that the largest HCC lesion measured 3.5 cm and that two additional lesions of approximately 2 cm were present, all confined to the right lobe, consistent with Barcelona Clinic Liver Cancer stage B disease. The patient underwent transarterial chemoembolization on January 27, 2025. However, subsequent CT showed tumor progression, and the disease was considered transarterial chemoembolization–refractory. At baseline (Day −1), liver function was preserved with a Child–Pugh score of 6 (trace ascites) and an ALBI score of −3.27. The platelet count was 223,000/μL, and the FIB‐4 index was 2.01. CT demonstrated mild splenomegaly; however, no definite varices were identified on endoscopy or CT. Systemic therapy with durvalumab plus tremelimumab was initiated on June 10, 2025 (Day 0).

Diarrhea developed on Day 5, and the patient presented for an unscheduled visit on Day 7. She was initially managed for grade 1 immune‐mediated colitis in an outpatient setting; however, on Day 9, she was found to have renal dysfunction and was urgently admitted with suspected grade 3 immune‐mediated colitis (Table 1). High‐dose corticosteroid therapy was promptly initiated (prednisolone equivalent 60 mg/day, 1 mg/kg/day), and lower gastrointestinal endoscopy with biopsy confirmed immune‐mediated colitis. Prednisolone was tapered to 50 mg/day on Day 22; however, because diarrhea worsened on Day 26, the dose was increased to 100 mg/day (2 mg/kg/day). Subsequently, the symptoms improved, and the steroid dose was tapered to 50 mg/day on Day 30, 40 mg/day on Day 37, 30 mg/day on Day 44, 20 mg/day on Day 51, 15 mg/day on Day 58, and 10 mg/day on Day 65. Thereafter, the colitis did not recur.

**TABLE 1:** Laboratory trends relative to initiation of durvalumab and tremelimumab.

	Day −1 pretreatment	Day 9 (admission)	Day 34	Day 48	Day 59	Day 61	Day 65	Day 69	Day 71	Day 73	Day 76
CRP (mg/dL)	0.4	4.2	3.5	2.2	2.1	2.2	4.6	3.4	2.6	1.6	1.2
Cr (mg/dL)	0.73	2.64	1.04	1.13	0.96	0.78	1.86	1.17	0.99	0.87	0.97
Alb (g/dL)	4.5	3.5	3.3	3.2	2.2	2.4	2.0	2.4	2.3	2.4	2.3
T‐Bil (mg/dL)	0.4	0.5	0.4	0.6	0.8	1.0	1.3	1.3	1.2	1.3	1.9
AST (U/L)	34	12	37	36	57	76	75	33	38	40	52
ALT (U/L)	29	12	49	49	102	123	119	67	62	68	77
Plt (10^4/μL)	22.3	38.4	20.5	20.0	13.4	13.4	10.6	6.4	4.5	5.2	7.6
PT (%)	84	61	NA	NA	82	81	51	79	67	67	64
Child‐Pugh	6	7	NA	NA	9	9	11	10	11	11	11
Modified ALBI score	−3.27	−2.61	−2.25	−2.05	−1.29	−1.23	−0.81	−1.15	−1.09	−1.15	−0.96
FIB‐4 index	2.01	0.77	1.83	1.83	2.99	3.63	4.61	4.47	7.61	6.62	5.54

*Note:* The PT and Child–Pugh scores were not available on Days 34 or 48. Day 0 indicates the start of durvalumab and tremelimumab therapy.

Abbreviations: Alb, albumin; ALT, alanine aminotransferase; AST, aspartate aminotransferase; Cr, creatinine; CRP, C‐reactive protein; FIB‐4 index, Fibrosis‐4 index; modified ALBI, modified albumin‐bilirubin score; NA, not available; Plt, platelets; PT, prothrombin time; T‐Bil, total bilirubin.

**FIGURE 1:**
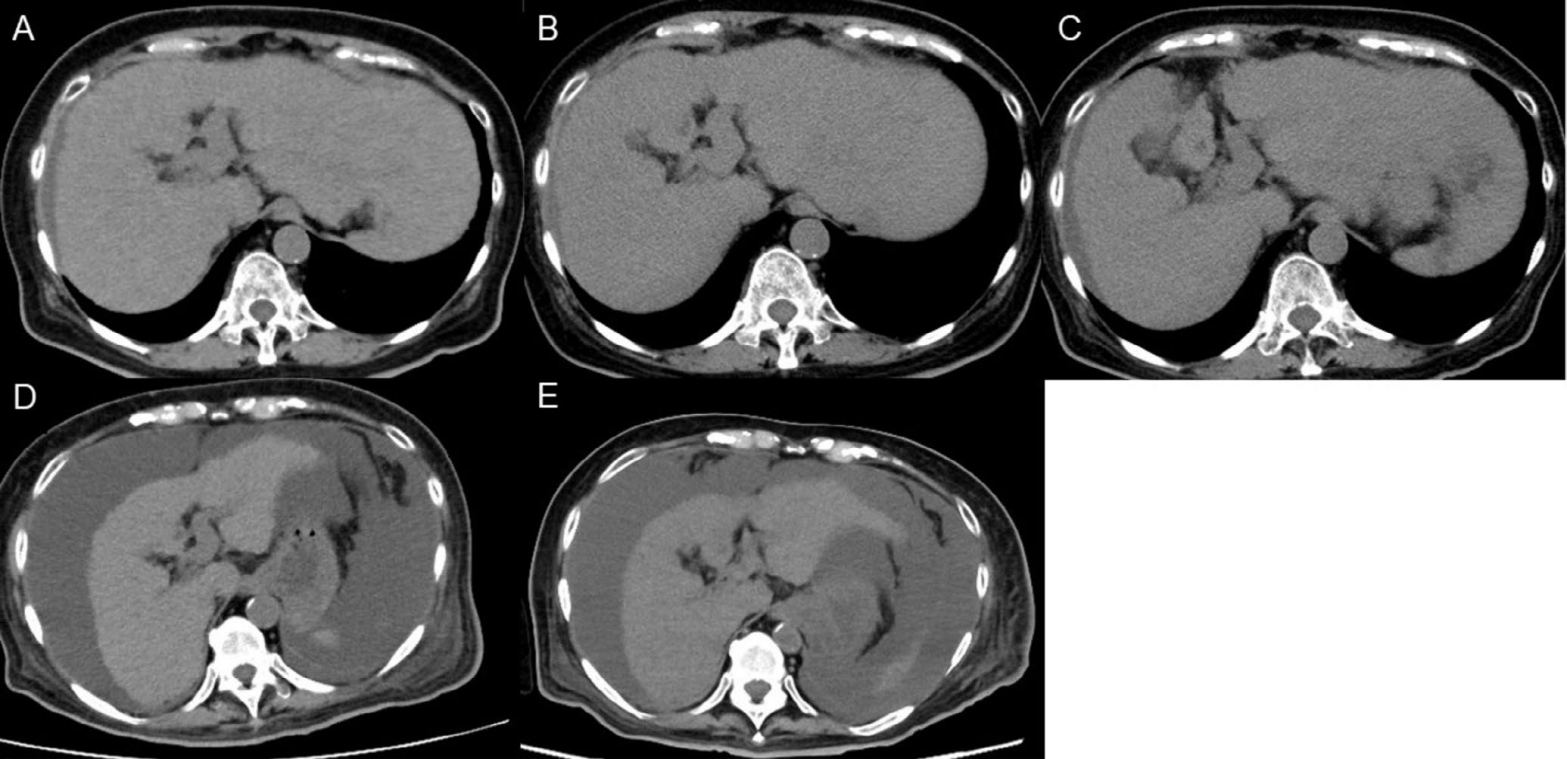
Non‐contrast axial computed tomographic images showing rapid progressive liver atrophy after durvalumab plus tremelimumab therapy. Representative non‐contrast axial computed tomographic images obtained at the portal vein bifurcation are shown at matched anatomical levels with identical window/level settings: (A) Day −1 (baseline), (B) Day 9 (admission), (C) Day 34, (D) Day 65 (marked atrophy became apparent), and (E) Day 80 (postmortem). Progressive parenchymal loss was evident over time without biliary dilatation. Day 0 indicates the initiation of durvalumab plus tremelimumab therapy on June 10, 2025.

**FIGURE 2:**
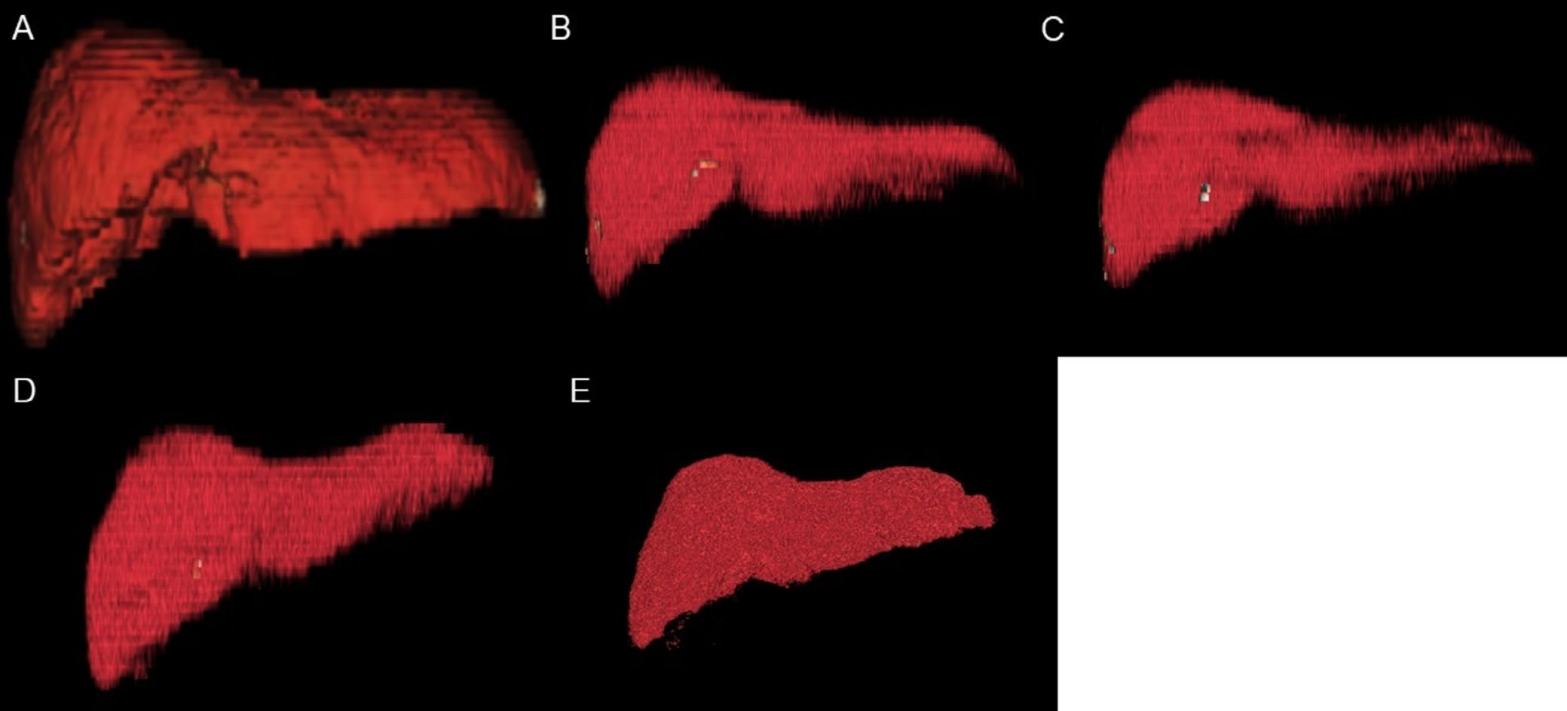
Three‐dimensional computed tomographic volumetry demonstrating progressive reduction in total liver volume after durvalumab plus tremelimumab. Three‐dimensional liver reconstructions generated from non‐contrast computed tomographic scans illustrate progressive liver shrinkage at (A) Day −1 (baseline), (B) Day 9 (admission), (C) Day 34, (D) Day 65 (marked atrophy), and (E) Day 80 (postmortem). Day 0 indicates the initiation of durvalumab plus tremelimumab therapy on June 10, 2025.

**FIGURE 3:**
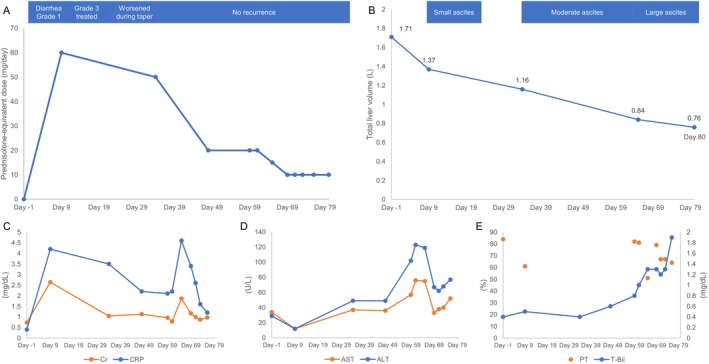
Integrated clinical time course after initiation of durvalumab plus tremelimumab. Day 0 indicates the initiation of durvalumab plus tremelimumab. All panels share a common time‐scaled *x*‐axis reflecting the actual elapsed days during the clinical course. (A) Clinical course of immune‐mediated colitis and prednisolone‐equivalent corticosteroid dosing. Diarrhea developed on Day 5, and grade 3 immune‐mediated colitis was treated on Day 9; symptoms worsened during steroid tapering, but no recurrence occurred thereafter. (B) Serial total liver volumes measured by non‐contrast CT volumetry and the progression of ascites (small, moderate, and large ascites) during the clinical course. Liver volume declined from 1.71 L at baseline (Day −1) to 0.76 L at death (Day 80). Volumetry was performed on non‐contrast computed tomographic images using SYNAPSE VINCENT (Fujifilm) with semi‐automated liver segmentation and manual contour correction. (C) Serial serum creatinine and C‐reactive protein levels. (D) Serial AST and ALT levels. (E) Serial total bilirubin and prothrombin time (PT). PT is shown as discrete time‐point measurements because it was not assessed at all time points.

Although the patient's gastrointestinal symptoms remained controlled, renal dysfunction and jaundice developed on Day 65, with elevated inflammatory markers, prompting CT evaluation. The patient remained afebrile, and CT showed no source of infection. CT and abdominal ultrasonography demonstrated no evidence of biliary obstruction, and imaging did not suggest vascular thrombosis. Because she developed dyspnea with pleural effusion, a cardiology consultation was obtained. Electrocardiography was performed, and no clinically significant arrhythmias were documented. High‐sensitivity troponin and creatine kinase‐MB levels were measured, with no significant abnormalities. Transthoracic echocardiography demonstrated preserved left ventricular systolic function (ejection fraction, 71.1%) without regional wall motion abnormalities. On this basis, acute de novo heart failure or acute circulatory collapse was not strongly suggested clinically at that time, and the pleural effusion was considered more consistent with fluid overload related to cirrhosis; therefore, diuretics were initiated. However, imaging revealed progressive hepatic atrophy (Figure 1). With intravenous fluid therapy, renal function improved by Day 69; however, liver failure progressed with increasing ascites, accompanied by worsening cholestasis and coagulopathy (Table 1). Given the progressive hepatic atrophy and decompensation, immune‐mediated hepatitis was considered; however, aminotransferase abnormalities remained modest, and the patient was already receiving high‐dose systemic corticosteroids for colitis. In parallel, non‐contrast CT volumetry demonstrated a stepwise decline in total liver volume (Figure 2). Volumetry was performed on non‐contrast CT images using SYNAPSE VINCENT (Fujifilm, Tokyo, Japan) with semi‐automated liver segmentation, followed by manual contour correction. The total liver volume decreased from 1.71 L at Day −1 to 1.37 L at Day 9, 1.16 L at Day 34, 0.84 L at Day 65, and 0.76 L at Day 80, representing an approximate 56% reduction from baseline (Figures 2 and 3). During the course, AST peaked at Common Terminology Criteria for Adverse Events version 6.0 grade 1, and ALT peaked at grade 2. Based on the available data, a clear temporal association between corticosteroid dose adjustments and trends in aminotransferase levels could not be confirmed; therefore, these trends were interpreted cautiously (Table 1). Despite supportive care, the patient died on Day 80. Postmortem CT demonstrated further progression of hepatic atrophy.

## 3 Differential Diagnosis

Rapid progression to liver failure prompted evaluation of alternative etiologies, as follows:

1. Infection (e.g., sepsis, cholangitis, intra‐abdominal infection): The patient remained afebrile, and CT imaging revealed no infectious focus.
2. Biliary obstruction (malignant or stone‐related): CT and abdominal ultrasonography revealed no bile duct dilatation; hence, biliary obstruction was unlikely.
3. Thrombotic disease (portal vein thrombosis or tumor thrombus): Imaging findings did not suggest vascular thrombosis.
4. HCC progression–related hepatic failure: The autopsy revealed a small tumor burden without lymph node or distant metastases, arguing against tumor progression as the primary cause of liver failure.
5. Circulatory failure/cardiogenic shock–related liver injury: Transthoracic echocardiography showed a preserved ejection fraction without regional wall motion abnormalities, and myocardial injury biomarkers were not elevated. Therefore, acute circulatory collapse was not strongly suggested at the relevant time point. Although acute circulatory collapse was not strongly suggested during the clinical evaluation (normal electrocardiography without clinically significant arrhythmias, non‐elevated high‐sensitivity troponin and creatine kinase‐MB, and preserved systolic function on echocardiography), autopsy demonstrated immune‐mediated myocarditis. Therefore, while myocarditis did not appear to be the primary driver of hemodynamic deterioration at the time of assessment, a contributory role of myocarditis in the overall clinical course cannot be fully excluded.

Overall, by integrating the clinical course, serial imaging, and autopsy findings, immune‐mediated hepatitis related to ICI therapy was considered the most likely cause of fatal liver failure.

## 4 Conclusion and Results

**FIGURE 4:**
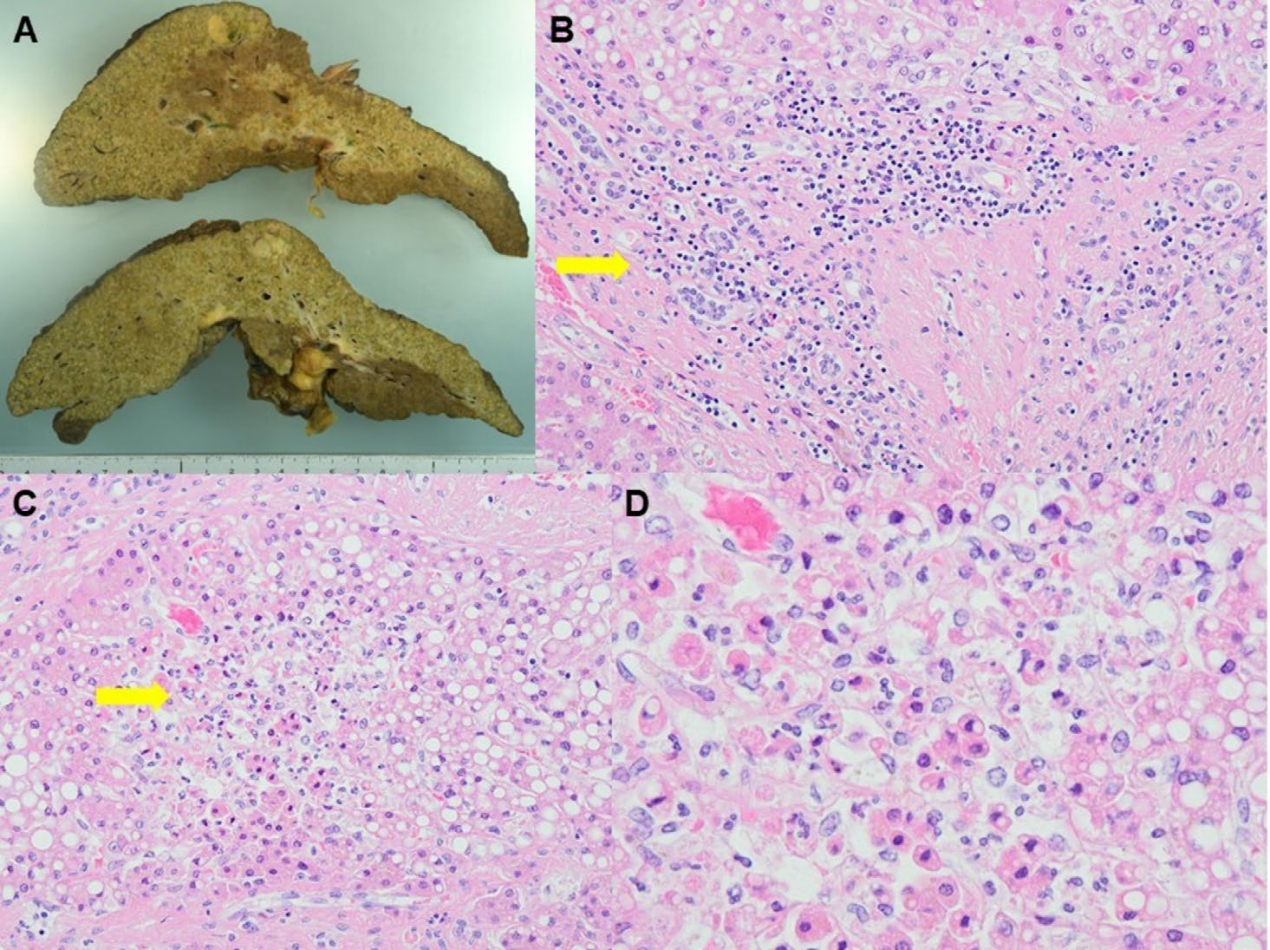
Autopsy liver findings showing marked atrophy with cirrhosis and lobular hepatitis with patchy necrosis. (A) Gross appearance of the liver demonstrating marked atrophy with multiple regenerative nodules; multiple well‐differentiated hepatocellular carcinoma nodules (≤ 2.0–2.5 cm) are present without vascular invasion. (B) Hematoxylin and eosin (H&E) staining shows lymphocyte‐predominant inflammatory cell infiltration extending from fibrous septa into pseudolobules (original magnification ×100). (C) Patchy hepatocellular necrosis within pseudolobules (H&E; original magnification ×200). (D) Higher magnification of a representative necrotic focus (H&E; original magnification ×400). Arrows indicate representative diffusely distributed patchy necrotic foci within pseudolobules and lymphocyte‐predominant inflammation extending from fibrous septa into pseudolobules.

**FIGURE 5:**
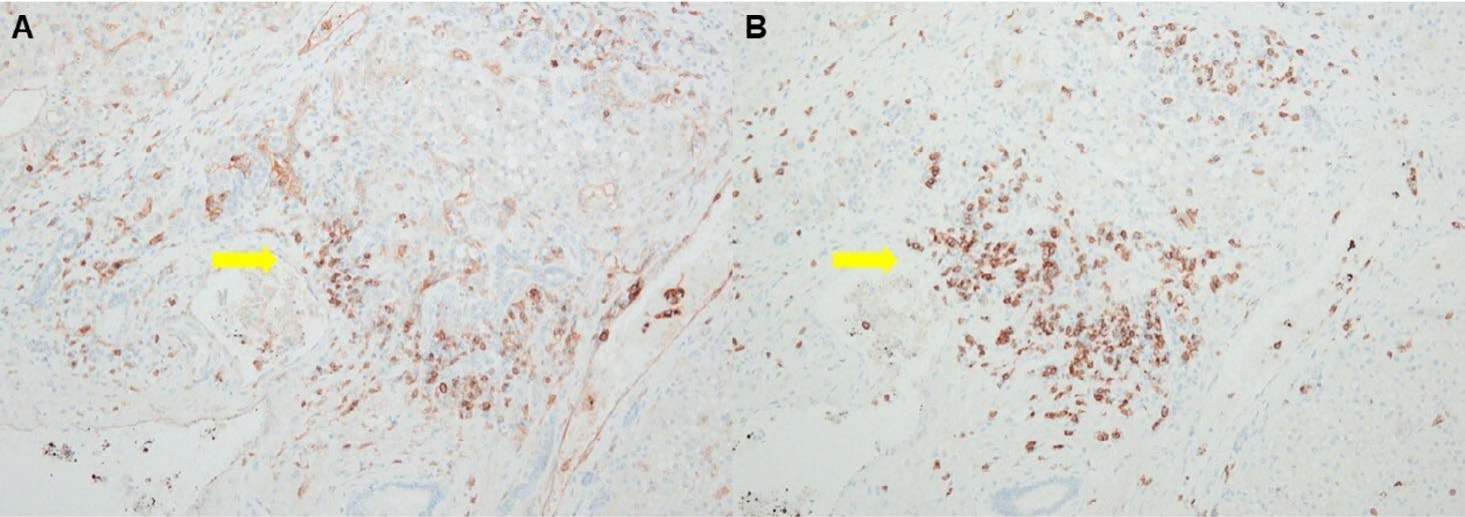
Immunohistochemistry demonstrating CD8‐predominant T‐cell infiltration in the liver. (A) CD4 immunostaining (original magnification ×100). (B) CD8 immunostaining (original magnification ×100). CD8 immunostaining highlights predominant CD8‐positive T‐cell infiltration within the fibrous septa and sinusoids, supporting immune‐mediated hepatitis. Arrows indicate representative positively stained lymphocytes.

To determine the cause of death, an autopsy was performed with the family's consent. The liver weighed 840 g and exhibited macronodular cirrhosis, lobular hepatitis, and patchy necrosis. No portal vein thrombosis or bile duct dilatation was observed (Figure 4). Moderate lymphocytic infiltration was present within the fibrous septa and sinusoids, and immunohistochemistry demonstrated predominantly CD3‐positive T cells with CD8 predominance. Histologically, patchy necrotic foci associated with lobular hepatitis were diffusely distributed throughout the liver within pseudolobules. Regenerative changes with clusters of small hepatocytes were more prominent toward the pseudolobular margins, and moderate lymphocytic infiltration was present in the fibrous septa and sinusoids. Although background steatotic liver disease was suggested, steatosis was only focally present and overt steatohepatitis features were limited in the autopsy specimens. Given the recent exposure to durvalumab plus tremelimumab, these findings were considered consistent with immune‐mediated hepatitis (Figure 5). At autopsy, multiple well‐differentiated HCC nodules measuring ≤ 2.0–2.5 cm were identified, all confined to the right lobe. No vascular invasion, lymph node metastases, or distant metastases were observed. A definite antitumor response attributable to durvalumab plus tremelimumab was not evident on pathological evaluation.

**FIGURE 6:**
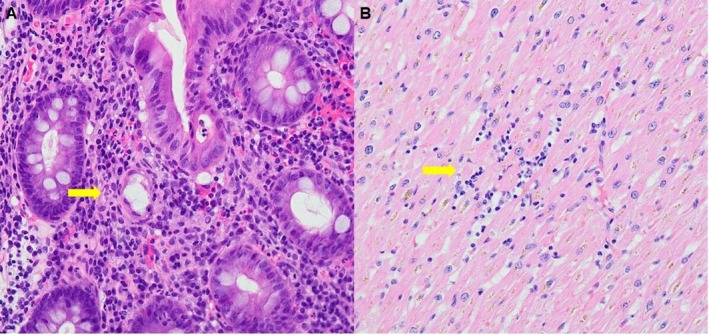
Autopsy findings consistent with immune‐mediated colitis and myocarditis. (A) Colonic mucosa shows lymphocyte‐predominant inflammation with crypt abscesses (hematoxylin and eosin [H&E]; original magnification ×100), consistent with immune‐mediated colitis. (B) The myocardium shows interstitial lymphocytic infiltration between myocardial fibers with associated myocyte injury (H&E; original magnification ×200), which is consistent with immune‐mediated myocarditis. Arrows indicate crypt abscesses (A) and interstitial lymphocytic infiltration (B).

The colon was edematous with scattered erosions. Histological examination revealed moderate‐to‐severe lymphocyte‐predominant mucosal inflammation with crypt abscesses, consistent with immune‐mediated colitis (Figure 6A).

The myocardium showed lymphocytic infiltration with CD8 predominance and associated myocyte injury, consistent with immune‐mediated myocarditis (Figure 6B).

Death was primarily attributed to immune‐mediated hepatitis, which led to liver failure with concurrent immune‐mediated colitis and myocarditis.

## 5 Discussion

This case is notable for the autopsy‐confirmed immune‐mediated hepatitis after durvalumab plus tremelimumab therapy, characterized by rapid progressive liver atrophy and fatal liver failure despite only modest aminotransferase elevation, whereas immune‐mediated colitis was controlled with high‐dose corticosteroids. In addition to serial non‐contrast CT imaging, CT volumetry provided objective quantification of progressive liver volume loss, strengthening the link between the clinical course and the pathological diagnosis.

Immune‐mediated hepatitis is typically suspected based on AST/ALT elevation, and severity grading often relies heavily on aminotransferases [11, 12, 13, 14]. However, in advanced cirrhosis, aminotransferase levels may underestimate the degree of hepatocellular injury. In the present case, AST/ALT fluctuated only mildly to moderately, and jaundice and coagulopathy developed later. Importantly, declining aminotransferase levels in progressive liver failure do not necessarily indicate resolution of hepatic inflammation; they may also reflect a reduction in viable hepatocyte mass as hepatic failure progresses. Therefore, the modest and/or declining AST/ALT levels in this case should not be interpreted straightforwardly as recovery. In contrast, CT volumetry demonstrated early and progressive liver volume loss, suggesting that surveillance strategies awaiting marked aminotransferase elevation may miss clinically significant and rapidly progressive immune‐mediated hepatitis in patients with cirrhosis. A “burnt‐out” phenotype in steatotic liver disease–related cirrhosis has been described and could have contributed to this clinicobiochemical discordance [15, 16]; however, a causal relationship cannot be established from a single case, and this phenomenon is more appropriately interpreted as a limitation of aminotransferase‐based monitoring in advanced cirrhosis.

The differential diagnosis of rapidly progressive liver failure includes infection, biliary obstruction, vascular thrombosis, tumor progression, and circulatory collapse [13]. In this case, infection was unlikely because the patient remained afebrile and imaging revealed no infectious source. CRP remained persistently elevated throughout the clinical course. Although infection is an important alternative explanation for systemic inflammation in patients with cirrhosis, we considered an infectious etiology unlikely because the patient remained afebrile and repeated imaging did not identify an infectious source and blood cultures were negative. We interpreted the sustained inflammatory response as more consistent with ongoing immune‐mediated inflammation and/or progressive tissue injury during the course—including immune‐mediated hepatitis and concomitant immune‐mediated myocarditis confirmed on autopsy—rather than an infectious process. In addition, systemic inflammation accompanying hepatic decompensation may have contributed to the elevated CRP levels. Biliary obstruction was not supported by the absence of bile duct dilatation on CT and ultrasonography, and imaging did not suggest vascular thrombosis. Transthoracic echocardiography revealed a preserved ejection fraction without regional wall motion abnormalities, and the clinical findings did not strongly suggest acute circulatory collapse. Moreover, autopsy demonstrated a limited, right‐lobe–confined tumor burden without vascular invasion or lymph node/distant metastasis, making tumor progression–related hepatic failure unlikely as the primary driver. The lobular pattern of hepatitis with diffusely distributed patchy necrosis and CD8‐predominant T‐cell infiltration supported immune‐mediated hepatitis in the clinical context and made alternative explanations such as obstructive cholestasis or active steatohepatitis less likely. Collectively, these findings suggest immune‐mediated hepatitis as the primary cause of fatal liver failure.

We acknowledge that prior locoregional therapy (TACE) and the presence of multifocal HCC could have contributed, at least in part, to hepatic parenchymal loss. However, in this case, the tumor burden remained limited and confined to the right lobe, whereas serial CT and CT volumetry suggested progressive atrophy that was not restricted to the tumor‐bearing lobe. In addition, the autopsy demonstrated lobular hepatitis with patchy necrosis and CD8‐predominant T‐cell infiltration consistent with immune‐mediated hepatitis. Taken together, these findings support immune‐mediated injury as the primary driver of progressive liver atrophy and fatal liver failure, although a partial contribution from prior TACE or tumor‐related factors cannot be completely excluded.

This case raises the practical question of how immune‐mediated hepatitis might be recognized earlier in patients with advanced cirrhosis. As progressive hepatic atrophy and decompensation became apparent (worsening ascites, jaundice, and coagulopathy), immune‐mediated hepatitis was considered in the differential; however, aminotransferase abnormalities remained modest and did not provide a strong biochemical signal of severe liver injury. Importantly, the patient was already receiving high‐dose systemic corticosteroids for immune‐mediated colitis, which is also the first‐line therapy for immune‐mediated hepatitis; therefore, escalation beyond corticosteroids was not pursued at that time. In retrospect, this case underscores that, in advanced cirrhosis, reliance on AST/ALT alone may delay recognition of clinically significant immune‐mediated liver injury. For cirrhotic patients receiving ICIs—particularly when another irAE has occurred—we recommend a lower threshold to suspect hepatic irAEs and a multidimensional monitoring strategy beyond aminotransferases, including bilirubin and coagulation parameters (PT/INR), albumin trends, signs of decompensation (ascites/encephalopathy), and, when feasible, repeat imaging with quantitative assessment (e.g., CT volumetry) to detect early progressive hepatic atrophy.

HCC has been reported as a clinical context associated with a higher risk of immune‐mediated liver injury during ICI therapy [17, 18]. Therefore, screening strategies that rely primarily on aminotransferases may be insufficient. When administering ICIs to patients with cirrhosis, a multidimensional assessment beyond AST/ALT should be considered, incorporating bilirubin, coagulation parameters, albumin trends, and signs of decompensation such as ascites. When feasible, quantitative imaging, such as CT volumetry, may provide an additional objective marker of progressive parenchymal loss. Importantly, the presence of one immune‐related adverse event (e.g., colitis) should lower the threshold for concurrent immune‐mediated hepatitis, even when the aminotransferase levels are modest.

This report has some limitations. Prothrombin time was not measured at all time points, preventing precise determination of when coagulopathy began. Nevertheless, the integration of autopsy findings (CD8‐predominant T‐cell infiltration with lobular hepatitis and patchy necrosis) with the clinical course and quantitative imaging supports the conclusion that immune‐mediated hepatitis can lead to fatal liver failure and may progress with minimal aminotransferase elevation in advanced cirrhosis. This case highlights the potential limitations of aminotransferase‐based surveillance in advanced cirrhosis and suggests the need for multidimensional monitoring strategies beyond AST/ALT.

## Author Contributions

**Risa Katsumata:** writing – review and editing. **Hikari Ishii:** writing – review and editing. **Kotaro Amano:** writing – review and editing. **Hidenobu Hara:** data curation, formal analysis, investigation, methodology, project administration, writing – original draft, writing – review and editing. **Ami Yoshinaga:** writing – review and editing. **Shoichi Yokobori:** writing – review and editing. **Kei Onodera:** writing – review and editing. **Jyunko Nemoto:** writing – review and editing. **Ryota Nakamura:** writing – review and editing. **Kazuomi Sakaki:** writing – review and editing. **Harujiro Yamamoto:** writing – review and editing. **Shiori Ito:** writing – review and editing. **Takehito Asakawa:** writing – review and editing. **Hiroaki Matsumoto:** writing – review and editing. **Moe Yamashita:** writing – review and editing. **Shinya Sakita:** writing – review and editing. **Jiro Kumagai:** writing – review and editing. **Kouhei Yoshino:** writing – review and editing.

## Funding

The authors have nothing to report.

## Ethics Statement

This report was approved by the Ethics Committee of Yokohama City Minato Red Cross Hospital (approval number: 2018–47 (1)).

## Consent

Written informed consent for publication of this case report (including autopsy findings) and any accompanying images was obtained from the patient's next of kin.

## Conflicts of Interest

The authors declare no conflicts of interest.

## Data Availability

The data that support the findings of this study are available from the corresponding author upon reasonable request.
